# Thromboxane prostanoid signaling in macrophages attenuates lymphedema and facilitates lymphangiogenesis in mice: TP signaling and lymphangiogenesis

**DOI:** 10.1007/s11033-023-08620-0

**Published:** 2023-08-04

**Authors:** Toshiaki Mishima, Kanako Hosono, Mina Tanabe, Yoshiya Ito, Masataka Majima, Shuh Narumiya, Kagami Miyaji, Hideki Amano

**Affiliations:** 1 Department of Cardiovascular Surgery, Kitasato University School of Medicine, Sagamihara, Japan; 2 Pharmacology, Kitasato University School of Medicine, Sagamihara, Kanagawa 252-0374 Japan; 3 Department of Molecular Pharmacology, Graduate School of Medical Sciences, Kitasato University, Sagamihara, Kanagawa 252-0374 Japan; 4 Department of Pharmacology, Kitasato University School of Medicine, 1-15-1 Kitasato, Minami-ku, Sagamihara, Kanagawa 252-0374 Japan; 5 Department of Medical Therapeutics, Kanagawa Institute of Technology, Atsugi, Kanagawa 243-0292 Japan; 6 Laboratory of Molecular and Cellular Physiology, Graduate School of Pharmaceutical Sciences, Kyoto University, Kyoto, 606-8507 Japan

**Keywords:** Lymphangiogenesis, Lymphedema, Macrophage, Thromboxane

## Abstract

**Background:**

Accumulating evidence suggests that prostaglandin E_2_, an arachidonic acid (AA) metabolite, enhances lymphangiogenesis in response to inflammation. However, thromboxane A_2_ (TXA_2_), another AA metabolite, is not well known. Thus, this study aimed to determine the role of thromboxane prostanoid (TP) signaling in lymphangiogenesis in secondary lymphedema.

**Methods and results:**

Lymphedema was induced by the ablation of lymphatic vessels in mouse tails. Compared with wild-type mice, tail lymphedema in *Tp*-deficient mice was enhanced, which was associated with suppressed lymphangiogenesis as indicated by decreased lymphatic vessel area and pro-lymphangiogenesis-stimulating factors. Numerous macrophages were found in the tail tissues of *Tp*-deficient mice. Furthermore, the deletion of TP in macrophages increased tail edema and decreased lymphangiogenesis and pro-lymphangiogenic cytokines, which was accompanied by increased numbers of macrophages and gene expression related to a pro-inflammatory macrophage phenotype in tail tissues. In vivo microscopic studies revealed fluorescent dye leakage in the lymphatic vessels in the wounded tissues.

**Conclusions:**

The results suggest that TP signaling in macrophages promotes lymphangiogenesis and prevents tail lymphedema. TP signaling may be a therapeutic target for improving lymphedema-related symptoms by enhancing lymphangiogenesis.

## Introduction

Damage to lymphatic vessels due to lymph node dissection during surgery or radiotherapy leads to the development of secondary lymphedema. In particular, secondary lymphedema occurs following lymphadenectomy and/or radiotherapy in patients with breast cancer [1, 2]. Lymphedema is characterized by swelling of the affected limb caused by retained extracellular fluid, which leads to chronic local inflammation and fibrosis [3, 4]. Currently, therapeutic approaches include manual lymphatic drainage and use of compression garments; however, these interventions can only transiently reduce or slow the progression of lymphedema [4, 5]. Thus, a better understanding of the pathology of lymphedema and having pharmacological therapeutic options are highly needed.

The lymphatic vascular system regulates the composition of fluid and volume in the interstitial space to maintain tissue fluid homeostasis [6–8]. Given that lymphedema is caused by interrupting lymphatic flow due to the surgical removal of lymph nodes and abnormal interstitial fluid clearance, the induction of new lymphatic vessel formation from the pre-existing lymphatic vasculature, i.e., lymphangiogenesis, to transport excessive fluids and recover lymphatic flow would be a therapeutic strategy for the management of secondary lymphedema.

Lymphangiogenesis is driven by pro-lymphangiogenic growth factors including vascular endothelial growth factor (VEGF)-C and VEGF-D secreted by immune cells [9]. VEGF-C and VEGF-D signals through vascular endothelial growth factor receptor 3 (VEGFR3) [10] expressed by lymphatic endothelial cells [11]. The VEGF-C/VEGFR3 axis is crucially involved in the normal development of lymphatics, postnatal lymphangiogenesis, and lymphatic repair [3].

Arachidonic acid (AA) metabolites, including prostaglandins (PGs), thromboxane (TX), and leukotrienes (LTs), are implicated in cardiovascular biology. Recent evidence suggests that AA metabolites regulate lymphangiogenesis in pathological conditions, including tumor growth, inflammation, and secondary lymphedema [12]. 5-lipoxygenase (5-LOX)-derived LTB_4_ was found to attenuate lymphangiogenesis and enhance lymphedema using a mouse tail model of lymphedema [13]. Indeed, 5-LOX-deficient mice or mice treated with a selective LTB_4_ inhibitor exhibit a reduction in tail swelling and enhancement of lymphangiogenesis. Accordingly, clinical studies using ketoprofen, a nonsteroidal anti-inflammatory drug that can inhibit 5-LOX more than COX (inhibit 5-LOX together with COX) have demonstrated that treatments could minimize subjective lymphedema-related symptoms but failed to reduce the lymphedema volume and improve swelling [2].

Conversely, a COX-2 inhibitor suppresses lymphangiogenesis and aggravates lymphedema by disturbing the lymphatic flow in the tail, suggesting that COX-2-derived PGE_2_ induces lymphangiogenesis and prevents lymphedema [14]. TXA_2_, another representative prostanoid, has been implicated in cardiovascular diseases because of its vasoconstriction and platelet aggregation activities [15]. Recently, we have shown that endogenous TXA_2_ facilitates lymphangiogenesis in the inflamed diaphragm induced by lipopolysaccharide (LPS) administration to the peritoneal cavity [16]. In this study, we demonstrated that thromboxane prostanoid (TP) receptor signaling accelerates the formation of new lymphatic vessels via increasing pro-lymphangiogenesis-stimulating factors by recruited macrophages. This finding allows us to hypothesize that TXA_2_/TP signaling also plays a critical role in lymphangiogenesis during secondary lymphedema. Therefore, this study examined whether endogenous TXA_2_ contributes to the stimulation of lymphangiogenesis to attenuate lymphedema in edematous mouse tails.

## Materials and methods

### Animals

Male receptor knockout (TP^−/−^) mice (8 weeks old) were generated as described previously [17]. Male C57BL/6 wild-type (WT) mice (8 weeks old) were purchased from CLEA Japan (Tokyo, Japan). The TP-floxed mice were generated as reported previously [18]. Mice with TP deletion in myeloid cells (C57BL/6 background) were generated by crossing TP-floxed homozygous mice with LysM-Cre mice, and WT littermates were used as control mice. In this study, LysMCre-TPfloxed mice were referred to as TP^Δmac^ mice and littermate control mice as control mice (Cont). All mice were housed at constant humidity (50% ± 5%) and temperature (25 °C ± 1 °C) on a light/dark cycle of 12/12 h and were given ad libitum access to water and food.

The experimental protocols were reviewed by the Institutional Animal Care and Use Committee of the Kitasato University School of Medicine and approved by the Dean of the Kitasato University School of Medicine (Approval no. 2022-060). All experimental studies were performed in accordance with the guidelines of the Science Council of Japan for animal experiment.

### Surgical model of tail lymphedema

A mouse tail model of lymphedema was created as described previously [19]. Briefly, mice were anesthetized by intraperitoneal (i.p.) injection of mixed anesthetic agents containing 4.0 mg/kg midazolam (Sandoz, a Novartis division, Basel, Switzerland), 0.75 mg/kg medetomidine hydrochloride (Nippon Zenyaku Kogyo, Fukushima, Japan), and 5.0 mg/kg butorphanol (Meiji Seika Pharma, Tokyo, Japan). To remove the dermal lymphatic vessels, a 5 mm-wide circumferential incision was made through the dermis 10 mm distal to the base. The effects of medetomidine were then reversed with an i.p. injection of 0.75 mg/kg atipamezole (Nippon Zenyaku Kogyo). The distal part of the incision was digitally photographed (Nikon COOLPIX 7900, Tokyo, Japan) weekly. The diameters of the maximum horizontal tails at the distal edge of the incision were measured. The rate of reduction in the tail diameter was calculated.

### Tissue sample preparation

For tissue collection, the animals were euthanized with isoflurane (Pfizer, Manhattan, NY, USA) 3 weeks after surgery. Tail tissues at the distal edge of the wounds were excised, and excised tissue samples were prepared for real-time reverse transcription-quantitative polymerase chain reaction (RT-qPCR) analysis and were cryosectioned for immunohistochemistry.

### Immunofluorescence staining

For immunofluorescent staining, excised tissue samples were fixed with periodate–lysine–paraformaldehyde fixative at 4 °C overnight. Following cryoprotection with 30% sucrose prepared in 0.1 M phosphate buffer (pH 7.2), the 50 μm-thick sections were incubated with 1% bovine serum albumin in a phosphate-buffered saline solution containing 0.5% Triton X-100 at room temperature for 1 h to block nonspecific binding. Sections were then incubated with rabbit anti-mouse TP (1:100; Almone Labs, Jerusalem, Israel), a rat anti-mouse CD68 (a glycoprotein highly expressed in macrophages) antibody (1:100; Bio-Rad Laboratories, Hercules, CA, USA), a rabbit anti-mouse lymphatic vessel endothelial hyaluronan receptor (LYVE-1) antibody (1:200; Abcam, Cambridge, MA, USA), a goat anti-mouse LYVE-1 antibody (1:100; BD Biosciences, Franklin Lakes, NJ, USA), a goat anti-mouse VEGF-C antibody (C-20, 1:50; Santa Cruz Biotechnology, Dallas, TX, USA), or a goat anti-mouse VEGF-D antibody (M-16, 1:50; Santa Cruz Biotechnology, Dallas, TX, USA) at 4 °C for 3 days. Then, the sections were incubated with the following secondary antibodies at 4 °C overnight: Alexa Fluor 488-conjugated donkey anti-rabbit IgG, Alexa Fluor 488-conjugated donkey anti-goat IgG, Alexa Fluor 594-conjugated donkey anti-rat IgG, or Alexa Fluor 594-conjugated donkey anti-goat IgG (1:200; all from Molecular Probes, Eugene, OR, USA). The nuclei were stained with 4’-6-diamidino-2-phenylindole (DAPI). Slides were observed, and all images were obtained under a fluorescence microscope (Biozero BZ-700 Series; Keyence, Osaka, Japan). After labeling, five optical fields (×400 magnification) were randomly selected, and the number of positive cells was counted.

The area of LYVE-1^+^ lymphatic vessels per field was measured using ImageJ (version 2.0; National Institutes of Health). The results are expressed as the percentage of lymphatic vessel areas. Lymphatic vessel diameters were also determined using ImageJ and expressed as the mean diameter of lymphatic vessels.

### In vivo microscopy

In vivo fluorescence microscopic imaging of the lymphatic microvasculature was performed as described previously [19]. Briefly, under anesthesia with mixed anesthetic agents containing 0.75 mg/kg medetomidine hydrochloride (Nippon Zenyaku Kogyo), 4.0 mg/kg midazolam (Sandoz, a Novartis division), and 5.0 mg/kg butorphanol), 10 µL of fluorescently labeled macromolecule (2000 kDa FITC-dextran; 2 mg/mL; Molecular Probes, Carlsbad, CA, USA) was injected intradermally into the tip of the distal portion of the tail. Mice were prepared for in vivo fluorescence microscopy 30 min after dye injection. The lymphatic microvasculature was observed under a fluorescence microscope (ECLIPSE FN1, upright type; Nikon, Tokyo, Japan). Microscopic images were captured with an objective lens (4×; Nikon, Tokyo, Japan). Images were acquired with a CCD camera (Evolve 512; Photometrics, Tokyo, Japan) and image analysis software (StreamPix, Norpix, Canada). The captured images were analyzed, and the area occupied by the fluorescent dye was determined using Image-Pro Plus (Media Cybernetics, Silver Spring, MD, USA). After the microscopic observation, the animals were euthanized with cervical dislocation.

### RT-qPCR analysis

Total RNA was extracted from tissues using RNAiso Plus (Takara Bio, Shiga, Japan). cDNA was constructed with 1 µg of total RNA using the ReverTra Ace qPCR RT Kit (TOYOBO Co., Ltd., Osaka, Japan). Quantitative PCR amplification was performed using TB Green Premix Ex Taq II (Tli RNaseH Plus; Takara Bio, Inc. Shiga, Japan). PCR amplification was performed with the following conditions: 95 °C for 10 s, followed by 40 cycles at 95 °C for 3 s and 60 °C for 20 s. The mRNA expression levels were calculated based on the comparative threshold cycle and normalized to glyceraldehyde-3-phosphate dehydrogenase (GAPDH) expression in each sample. The fold change relative to 0 weeks was calculated. The primer sequences are listed in Table 1.

**Table 1:** The primers used for reverse transcription and quantitative PCR reactions

Gene	Forward primer sequence (5’-3’)	Reverse primer sequence (5’-3’)
*TP*	CCTCCTGCTCAACACCGTTAG	CTGAACCATCATCTCCACCTC
*TXS*	GGATTCTGCCCAATAAGAACC	GAAGTCTCTCCGCCTCTCTTC
*VEGF-C*	TCTGTGTCCAGCGTAGATGAG	GTCCCCTGTCCTGGTATTGAG
*VEGF-D*	CCTATTGACATGCTGTGGGAT	GTGGGTTCCTGGAGGTAAGAG
*VEGFR3*	GGAAGGCTCTGAAGATAAAGG	ACAGAAGATGAGCAGGAGGAG
*LYVE-1*	GCTCTCCTCTTCTTTGGTGCT	TGACGTCATCAGCCTTCTCTT
*Prox1*	GTTCTTTTACACCCGCTACCC	ACTCACGGAAATTGCTGAACC
*CCL2*	CGGAACCAAATGAGATCAGAA	TTGTGGAAAAGGTAGTGGATG
*CCR2*	TTACCTCAGTTCATCCACGGC	CAAGGCTCACCATCATCGTAG
*TNFα*	TCTTCTCATTCCTGCTTGTGG	GATCTGAGTGTGAGGGTCTGG
*IL-1β*	TACATCAGCACCTCACAAGCA	CCAGCCCATACTTTAGGAAGA
*MR*	TTTGTCCATTGCACTTTGAGG	TGCCAGGTTAAAGCAGACTTG
*IL-10*	CGGAAATGATCCAGTTTTACC	TGAGGGTCTTCAGCTTCTCAC
*GAPDH*	ACATCAAGAAGGTGGTGAAGC	AAGGTGGAAGAGTGGGAGTTG

### Cell preparation and culture

To generate bone marrow (BM)-derived macrophages, BM cells were isolated from the femurs and tibias of 8-week-old male mice. BM cells were cultured in 6-well plates (1.0 × 10^6^ cells per well) and maintained in Roswell Park Memorial Institute (RPMI) 1640 medium (Gibco, Thermo Scientific, Waltham, MA, USA) containing 10% fetal calf serum and 20 ng/mL macrophage colony-stimulating factor (BioLegend, San Diego, CA, USA) as described previously [20]. On day 7, BM-derived macrophages were stimulated with LPS (10 ng/mL; Sigma-Aldrich, St. Louis, MO, USA) and recombinant murine interferon-gamma (IFN-γ) (20 ng/mL; BioLegend) to polarize toward a pro-inflammatory macrophage phenotype or recombinant murine interleukin (IL)-4 (20 ng/mL; BioLegend) and to polarize toward a reparative phenotype in RPMI 1640 medium for 18 h. The cultured BM-derived macrophages were then harvested and homogenized in RNAiso Plus (Takara Bio), and the mRNA levels were measured by RT-qPCR.

### Statistical analysis

All results are expressed as the mean ± standard deviations. All statistical analyses were performed using GraphPad Prism version 8 (GraphPad Software, La Jolla, CA, USA). Data were compared between two groups using unpaired two-tailed Student t-tests and between multiple groups using one-way analyses of variance, followed by Tukey’s post hoc tests. P-values of < 0.05 were considered statistically significant.

## Results

### TP^−/−^ mice display exacerbated tail lymphedema

**Fig. 1:**
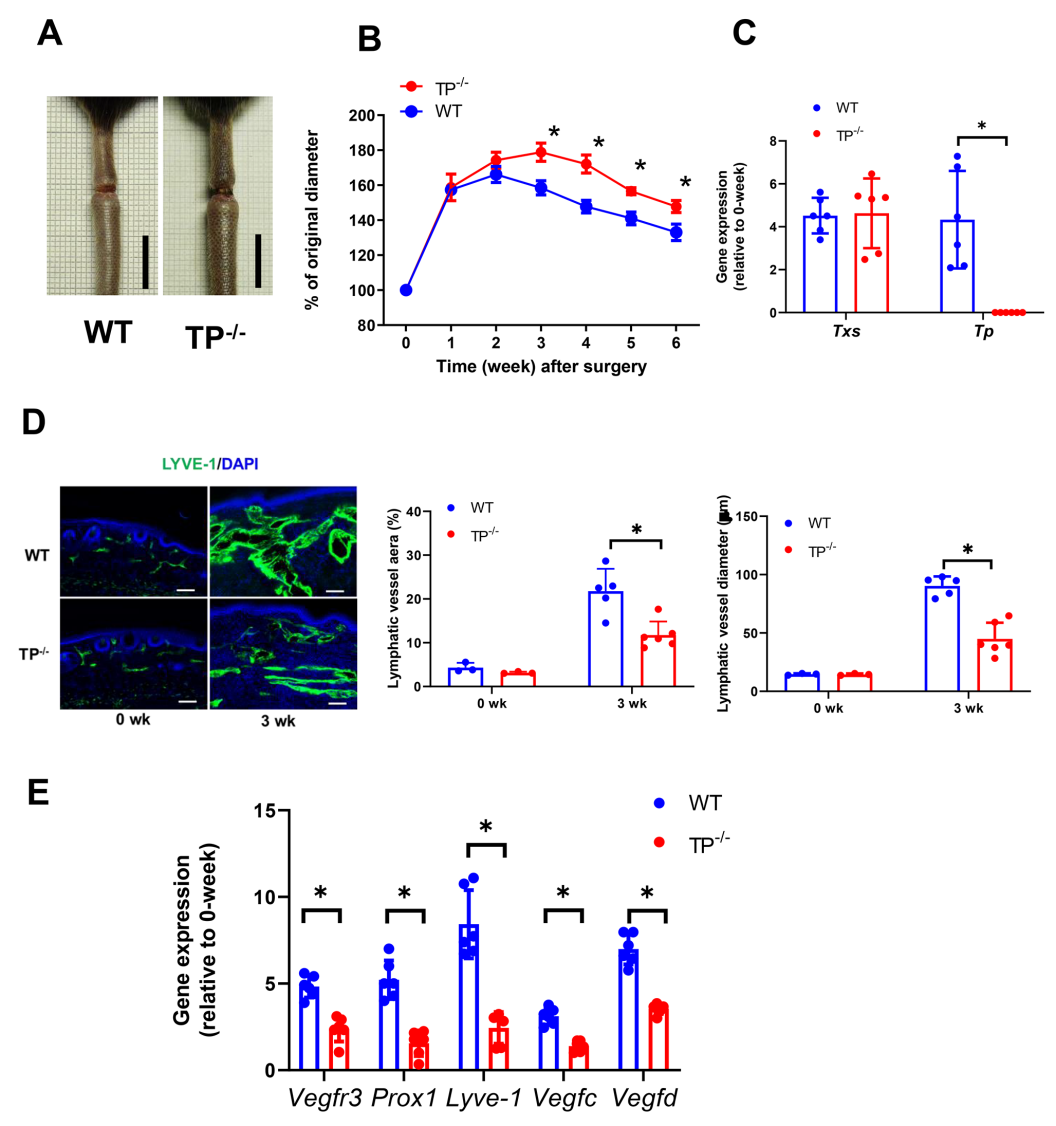
Aggravated tail lymphedema and suppressed lymphangiogenesis in TP^−/−^ mice (**A**) Representative photographs of mouse tails 3 weeks after surgery in WT mice and TP^−/−^ mice. Scale, 1 cm (**B**) Changes in the tail diameters of WT and TP^−/−^ mice after surgery (**C**) mRNA levels of *Txs* and *Tp* at the distal edge of the wound 3 weeks after surgery. Data are expressed as the mean ± SD. *P < 0.05 (**D**) Immunofluorescence staining of LYVE-1 (green) in the distal wound regions from WT and TP^−/−^ mice 0 and 3 weeks after surgery. Cell nuclei were stained with DAPI (blue). Scale bar: 100 μm. The lymphatic vessel area percentage and diameter of tail tissues from WT and TP^−/−^ mice 0 and 3 weeks after surgery. Data are expressed as the mean ± SD. *P < 0.05 (**E**) mRNA expression levels of *Vegfr3, Prox1, Lyve-1, Vegfc*, and *Vegfd* in tail tissues from WT and TP^−/−^ mice 3 weeks after surgery. Data are expressed as the mean ± SD. *P < 0.05

To determine the role of TP signaling in tail edema, tail lymphatic vessels of WT mice and TP^−/−^ mice were resected (Fig. 1A). The surgical removal of the lymphatic vessels resulted in apparent lymphedema (Fig. 1A). In WT mice, the tail diameter was increased after surgery and reached its maximum 2 weeks after surgery. Then, the diameter decreased gradually; however, the tail diameter 6 weeks after surgery was still 40% larger than the basal level (Fig. 1B). The time course of changes in the tail diameter in TP^−/−^ mice appeared to be essentially the same as those in WT mice, and the tail swellings peaked 3 weeks after surgery. In WT mice, the mRNA expression levels of *Txs* and *Tp* in the regions distal to the tail wound 3 weeks after surgery were increased approximately 4-fold relative to those 0 week after surgery. In TP^−/−^ mice, mRNA levels of *Txs* 3 weeks after surgery were increased approximately 4-fold relative to those 0 week after surgery, but not TP mRNA levels did not increase as expected (Fig. 1C). These results suggest that TP signaling reduces tail lymphedema.

### TP^−/−^ mice suppress lymphangiogenesis in the tail lymphedema

Given the improving lymphedema through TP signaling, we next sought to determine the effect of TP signaling on corresponding lymphangiogenesis by assessing the morphological alterations in the tail tissue Sect. 3 weeks after surgery. Cross-sections of the region distal to the wound were stained with an antibody against LYVE-1, a lymphatic vessel marker (Fig. 1D). Immunofluorescence demonstrated the presence of LYVE-1-positive lymphatic structures in both mouse types; however, few LYVE-1-positive lymphatic vessels were found in TP^−/−^ mice as compared with those in WT mice. Quantitative analysis of the lymphatic vessel area confirmed that lymphedema-induced lymphangiogenesis was 50% lower in TP^−/−^ mice than in WT mice (Fig. 1D). In addition, enlarged LYVE-1^+^ lymphatic vessels were observed in WT mice. The diameter of lymphatic vessels in WT mice was also larger than those in TP^−/−^ mice (Fig. 1D).

### mRNA expression related to lymphatic endothelial cells and pro-lymphangiogenic factors

Then, we determined the gene expression related to lymphatic endothelial cells and lymphangiogenesis-stimulating factors in tail tissues at the distal edge of the wound. Compared with WT mice, TP^−/−^ mice displayed reductions in mRNA levels of *Vegfr3, Lyve-1*, and *Prox1*, a marker highly expressed in lymphatic endothelial cells in tail tissues 3 weeks after surgery (Fig. 1E). In addition, the mRNA expression levels of *Vegfc* and *Vegfd* were lower in TP^−/−^ mice than in WT mice. These results suggest that the enhanced lymphangiogenesis in the tail tissues is associated with increased expression of lymphatic endothelial cells and lymphangiogenesis-stimulating factors, which is dependent on TP signaling.

### Accumulation of macrophages in tail tissues

**Fig. 2:**
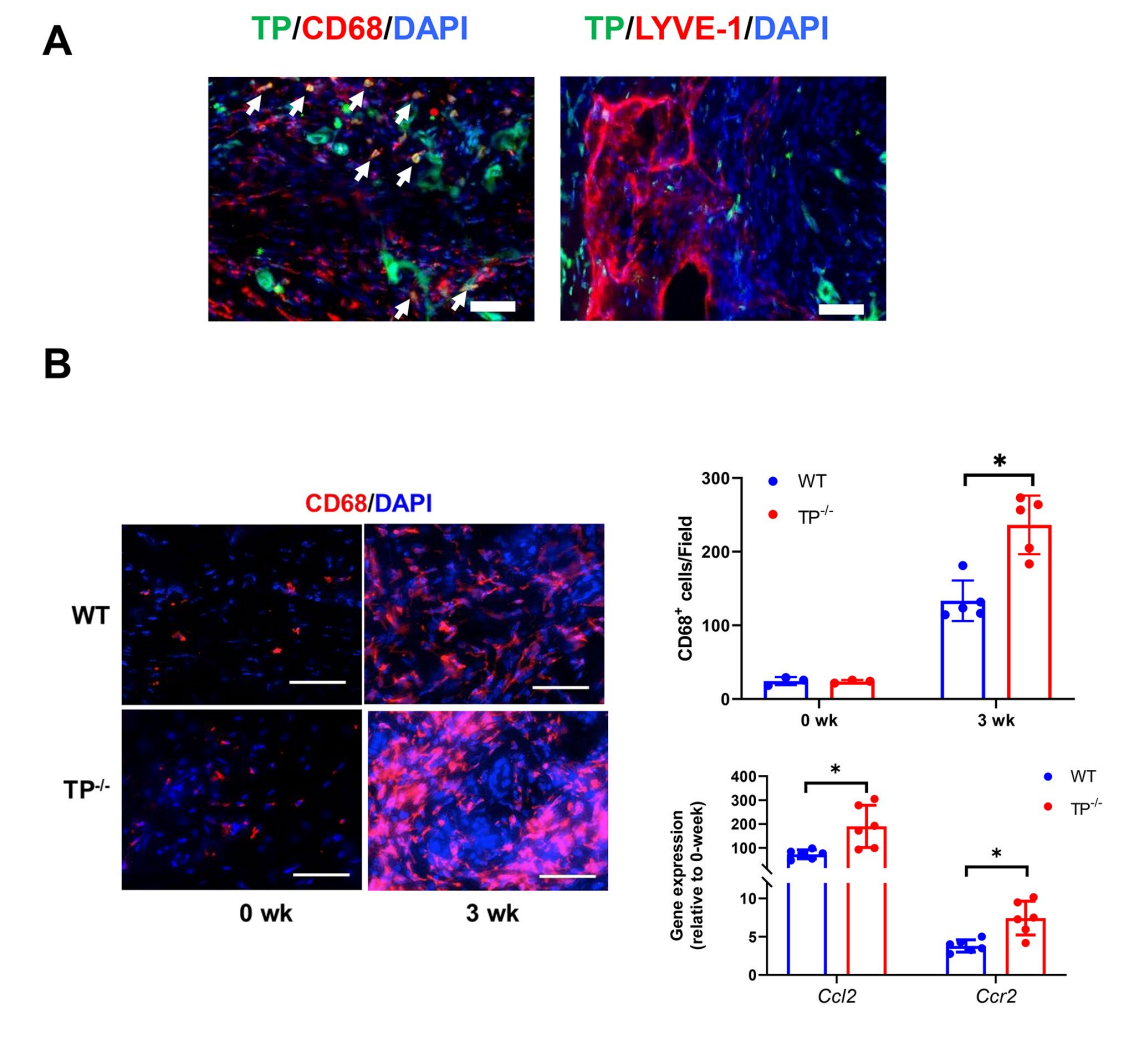
Accumulation of macrophages in edematous tail tissues (**A**) Double immunostaining of TP (green) and CD68 (red) or LYVE-1 (red) in tail tissues from WT mice 3 weeks after surgery. Cell nuclei were stained with DAPI (blue). White arrows indicate double-positive cells. Scale bar: 50 μm (**B**) CD68 immunostaining (red) in tail tissues from WT and TP^−/−^ mice 0 and 3 weeks after surgery. Cell nuclei were stained with DAPI (blue). Scale bar: 50 μm. Number of CD68^+^ cells in WT and TP^−/−^ mice. The mRNA expression levels of *Ccl2* and *Ccr2* in tail tissues from WT and TP^−/−^ mice 3 weeks after surgery. Data are expressed as the mean ± SD. *P < 0.05

Given that TP signaling contributes to lymphangiogenesis, we investigated the cellular source of TP during the healing of lymphedema in the tail tissues of WT mice. Double immunofluorescence analysis revealed that TP expression in the regions distal to the wounds co-localized with CD68^+^ cells (macrophages), but not LYVE-1^+^ lymphatic vessels (Fig. 2A). These results suggest that macrophages are involved in lymphangiogenesis through TP signaling and that TP signaling does not directly affect lymphatic vessels to proliferate in tail tissues.

Furthermore, macrophage recruitment has been implicated in lymphangiogenesis after surgical induction of lymphedema in the tail. Then, we determined the number of CD68^+^ cells 3 weeks after surgery. Immunofluorescence analysis demonstrated substantial accumulation of CD68^+^ cells in the regions distal to the wound in TP^−/−^ mice, and the numbers of CD68^+^ cells in TP^−/−^ mice were larger than those in WT mice (Fig. 2B). In addition, the expression levels of mRNA-encoding chemokine (C-C motif) ligand 2 (CCL2) and C-C chemokine receptor type 2 (CCR2) in TP^−/−^ mice were higher than those in WT mice (Fig. 2B).

### Deficient TP signaling in macrophages exacerbates tail lymphedema

**Fig. 3:**
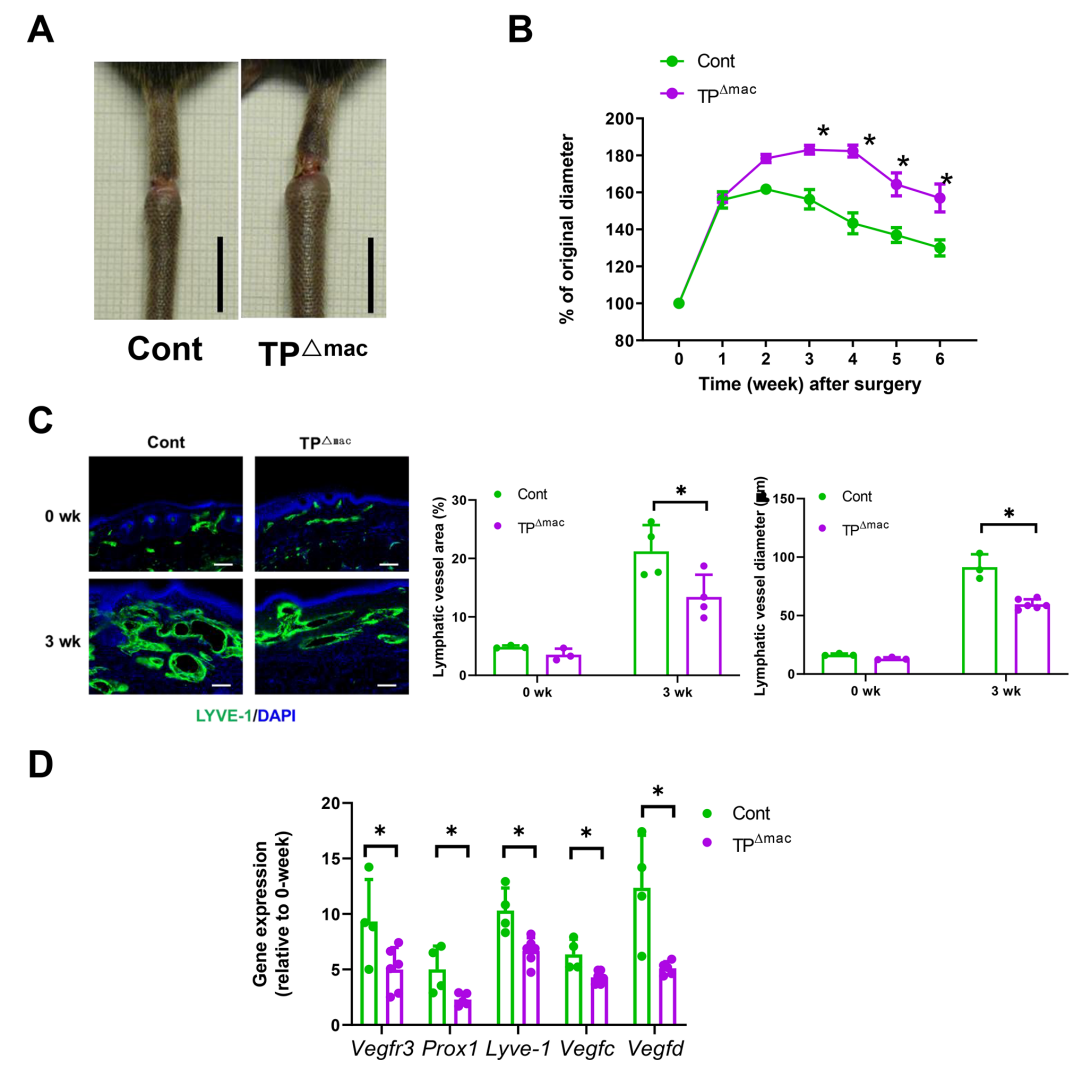
Aggravated tail lymphedema and suppressed lymphangiogenesis in TP^Δmac^ mice> (**A**) Typical appearance of lymphedema 3 weeks after surgery in control and TP^Δmac^ mice. Scale, 1 cm. (**B**) Changes in the tail diameters of control and TP^Δmac^ mice after surgery. (**C**) Immunofluorescence staining of LYVE-1 (green) in the distal wound regions from WT and TP^−/−^ mice 0 and 3 weeks after surgery. Cell nuclei were stained with DAPI (blue). Scale bar: 100 μm. The lymphatic vessel area percentage and diameter of tail tissues from control and TP^Δmac^ mice 0 and 3 weeks after surgery. Data are expressed as the mean ± SD. *P < 0.05 (**D**) mRNA expression levels of *Vegfr3, Prox1, Lyve-1, Vegfc*, and *Vegfd* in tail tissues from control and TP^Δmac^ mice 3 weeks after surgery. Data are expressed as the mean ± SD. *P < 0.05. Cont, controls

The above results suggest that the inhibition of TP signaling suppressed lymphangiogenesis and aggravated tail lymphedema by the accumulation of macrophages in the tail tissues. To further confirm the role of TP signaling in macrophages in tail lymphedema, TP^Δmac^ mice or control mice were subjected to surgical secondary lymphedema. Compared with control mice, TP^Δmac^ mice had enhanced tail lymphedema (Fig. 3A) and larger tail diameters after the induction of lymphedema (Fig. 3B). Lymphangiogenesis as indicated by the area, and the diameter of lymphatics was attenuated in TP^Δmac^ mice as compared with that in control mice (Fig. 3C). Reduced lymphangiogenesis was associated with decreased gene expression of lymphatic endothelial cells (*Vegfr3*, *Lyve-1*, and *Prox1*) and lymphangiogenesis-stimulating factors (*Vegfc* and *Vegfd*) in TP^Δmac^ mice (Fig. 3D).

### Pro-lymphangiogenic cytokines from macrophages

**Fig. 4:**
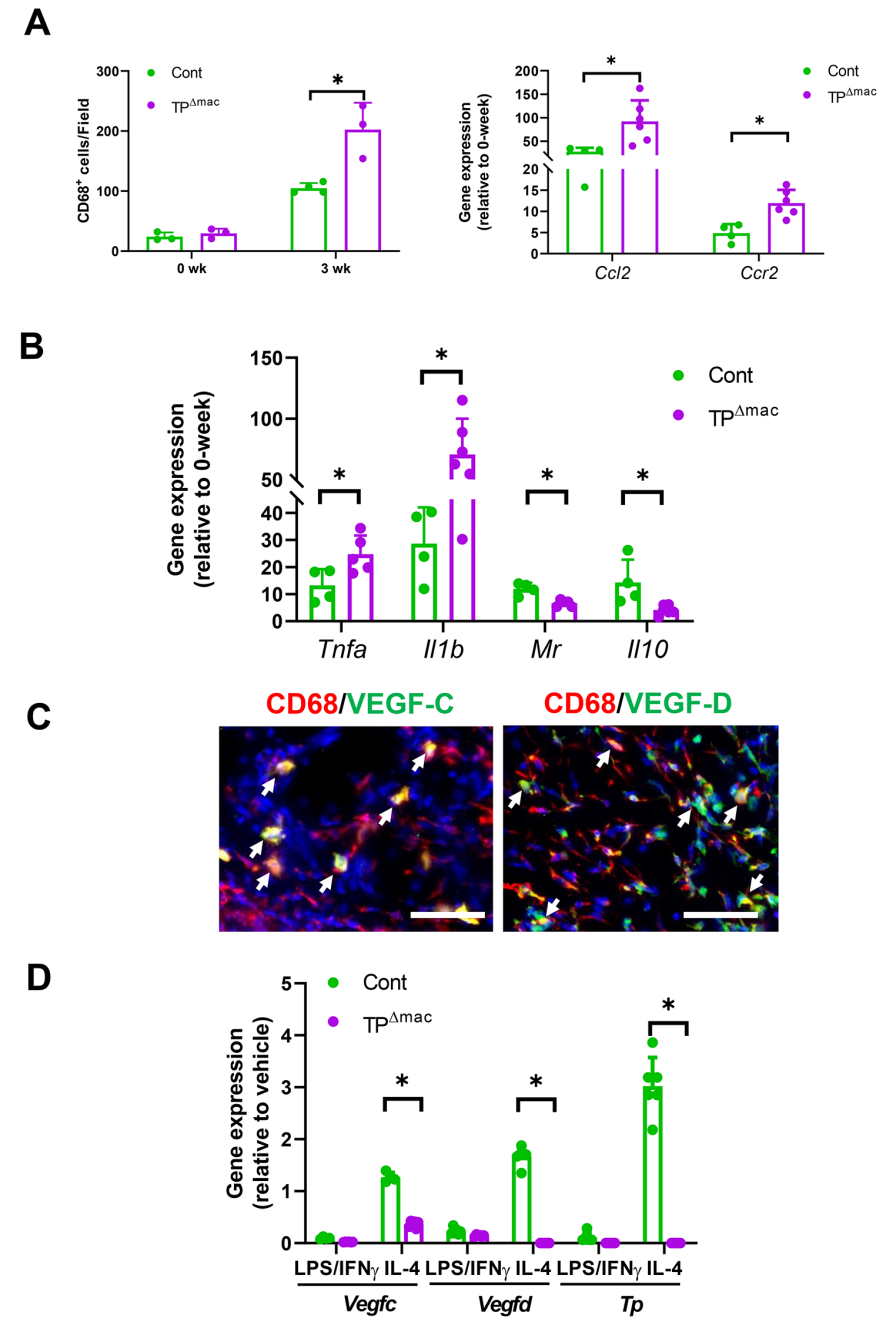
Accumulation of macrophages in the tail tissues and pro-lymphangiogenic factors in macrophages (**A**) Number of CD68^+^ cells and mRNA expression levels of *Ccl2* and *Ccr2* in tail tissues from control and TP^Δmac^ mice 0 and 3 weeks after surgery. Data are expressed as the mean ± SD. *P < 0.05. (**B**) mRNA expression levels of pro-inflammatory macrophage phenotype-related genes, including *Tnfa* and *Il1b* and reparative macrophage phenotype-related genes, including *Mr* and *Il10*, in tail tissues from control and TP^Δmac^ mice 3 weeks after surgery. Data are expressed as the mean ± SD. *P < 0.05. (**C**) Double immunostaining of CD68 (red) and VEGF-C (green) or VEGF-D (green) in tail tissues from control and TP^Δmac^ mice 3 weeks after surgery. Cell nuclei were stained with DAPI (blue). White arrows indicate double-positive cells. Scale bars, 50 μm. (**D**) mRNA expression levels of *Vegfc*, *Vegfd*, and *Tp* in cultured macrophages from control and TP^Δmac^ mice stimulated with LPS/IFN-γ or IL-4. Data are expressed as the mean ± SD. *P < 0.05. Cont, controls

Moreover, we evaluated the recruitment of macrophages in the tail tissues. CD68^+^ cells in TP^Δmac^ mice were more accumulated than those in control mice, which was accompanied by increased expression levels of *Ccl2* and *Ccr2* (Fig. 4A). These were also associated with increased mRNA levels related to pro-inflammatory macrophages including tumor necrosis factor alpha (*Tnfa)* and *Il1b* and with decreased levels related to anti-inflammatory macrophages including mannose receptor (*Mr)* and *Il10* in tail tissues of TP^Δmac^ mice (Fig. 4B). These results suggest that the accumulation of anti-inflammatory macrophages was suppressed in the lymphangiogenesis of TP^Δmac^ mice, resulting in tail lymphedema.

Moreover, we examined whether macrophages are the cellular sources of VEGF-C and VEGF-D. Immunofluorescence demonstrated that VEGF-C and VEGF-D were co-localized with CD68^+^ cells, indicating that macrophages produce pro-lymphangiogenesis-stimulating factors, including VEGF-C and VEGF-D (Fig. 4C). In addition, the mRNA expression levels of *Vegfc* and *Vegfd* were enhanced in macrophages from control mice stimulated with IL-4, but not with LPS/IFN-γ (Fig. 4D). These results suggest that macrophages that polarized toward a reparative phenotype produce VEGF-C and VEGF-D. In addition, the gene levels of *Tp* were increased in control-derived macrophages stimulated with IL-4, but not with LPS/IFN-γ, while the *Tp* gene levels did not change in TP^Δmac^ mice–derived macrophages stimulated with LPS/IFN-γ or IL-4. These results indicated that reparative macrophages, but not pro-inflammatory macrophages strongly expressed *Tp* gene levels, and enhanced expression of *Tp* was accompanied by polarization of macrophages toward a reparative phenotype, but not toward a pro-inflammatory phenotype. These results suggest that TP may be important for the polarization of macrophages toward a reparative phenotype.

### Dysfunction of lymphatic transport in the tail tissues in macrophages with specific TP deficiency

**Fig. 5:**
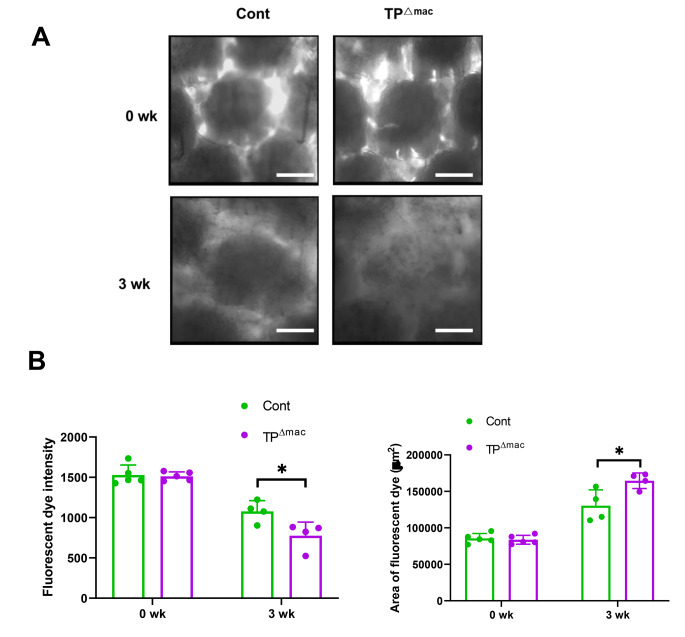
Lymphatic microcirculation in the regions distal to the wound tissues in the tails of control and TP^**Δmac**^ **mice** (**A**) Representative in vivo micrographs showing the tail lymphatic microvasculature 0 and 3 weeks after surgery. Scale bar, 50 μm. (**B**) Fluorescent intensity and area stained with fluorescent dye in control and TP^Δmac^ mice 0 and 3 weeks after surgery. Data are expressed as the mean ± SD. *P < 0.05. Cont, controls

To assess the function of the lymphatics, the fluorescent dye FITC-dextran was injected intradermally at the distal portion of the tail 3 weeks after surgery. In vivo fluorescence microscopy studies demonstrated that intact mouse tail skin displayed a well-defined hexagonal network of dermal lymphatics, indicating normal lymphatic flow and structures (Fig. 5A). The fluorescent dye intensity was remarkably low in both TP^Δmac^ and control mice. In control mice, the fluorescent dye was extravasated into the adjacent interstitial space, whereas in TP^Δmac^ mice, the boundary of the lymphatic microstructure appeared to be indistinct, indicating that lymphatic transport was compromised. The area occupied by the accumulated fluorescence intensity in TP^Δmac^ mice was larger than that in control mice (Fig. 5B), whereas the fluorescence intensity in the area in TP^Δmac^ mice was lower than that in control mice. These results suggest that TP signaling in macrophages is involved in the transportation and drainage of lymphatic fluids in tail lymphedema.

## Discussion

This study examined the role of endogenous TP signaling in lymphedema and lymphangiogenesis using a murine tail lymphedema model. Our data showed that surgical ablation of lymphatic vessels resulted in tail lymphedema, and the inhibition of TP signaling sustained lymphedema and suppressed lymphangiogenesis, which were associated with the accumulation of macrophages in the injured sites. TP-deficient signaling in macrophages increased tail lymphedema and recruitment of pro-inflammatory macrophages, attenuated lymphangiogenesis and lymphangiogenesis-stimulating cytokines, and impaired lymphatic fluid transport. These results suggest that TP signaling in macrophages improved tail lymphedema by inducing lymphangiogenesis. The specific activation of TP signaling in macrophages may be a therapeutic target for secondary lymphedema.

Accumulating evidence shows that lipid mediators, including LTs and prostanoids, play important roles in promoting lymphangiogenesis in several pathological conditions. The inhibition of LTB_4_ synthesis attenuated surgically induced tail lymphedema [13]. We have shown that the COX-2-derived PGE_2_ pathway promotes lymphangiogenesis in experimental models of wound healing [21, 22], peritonitis [18], tumor growth [23], and tail edema [14]. Recruited macrophages are involved in the formation of new lymphatic vessels by secreting pro-lymphangiogenic growth factors VEGF-C and VEGF-D. In addition to PGE_2_, we found that TXA_2_/TP signaling is responsible for the development of lymphangiogenesis in the inflamed diaphragm [16]. TP^−/−^ mice showed reduced LPS-induced lymphangiogenesis in diaphragm tissues and drainage function from the peritoneal cavity. The results are consistent with our recent findings, demonstrating that TP^−/−^ mice suppressed improvement in tail edema and reduced lymphatic transport by lymphatic vessels in tail edema. Current studies have also shown that TP-deficient signaling suppressed lymphangiogenesis, as indicated by the attenuated formation of LYVE-1^+^ lymphatic vessel-like structures and lymphatic endothelial cell markers.

TP signaling has been implicated not only in vascular tone, but also angiogenesis during tumor growth [24], hindlimb ischemia [25], and tissue repair [26]. The stimulation of TP signaling in lung cancer cells increases VEGF-A secretion through intracellular downstream activities, including extracellular signal-regulated kinase and protein kinase A [24]. In addition to angiogenesis, TP signaling exhibits pro-lymphangiogenic properties in LPS-induced inflammatory tissues [16]. The stimulation of TP signaling also increases VEGF-C and VEGF-D in macrophages in inflamed diaphragm tissues, which is consistent with our results. VEGF-C and VEGF-D expression levels in cultured BM-derived macrophages were upregulated in a TP signaling-dependent manner. Although the exact molecular mechanisms by which TP signaling enhances VEGF-C and VEGF-D in macrophages remain unknown, the accumulation of cyclic AMP via TP signaling may possibly activate cyclic AMP-responsive element-binding protein to drive the gene transcription of VEGF-C [27] and VEGF-D [28].

Our data further support the hypothesis that TP signaling in macrophages contributes to lymphedema reduction and lymphangiogenesis improvement. Macrophages of TP signaling-deficient mice exhibited significant tail edema after surgical induction of lymphedema compared with those of control mice. The transport of fluorescent dye injected to the distal regions of the surgical wound was disrupted in the macrophages of TP signaling-deficient mice as compared with those of control mice. Furthermore, lymphangiogenesis and pro-lymphangiogenic growth factor VEGF-C and VEGF-D were reduced in the macrophages of TP signaling-deficient mice. These results suggest that TP signaling in macrophages exerts pro-lymphangiogenic effects. Accumulated macrophages in the lymphedematous tissues are involved in the promotion of lymphangiogenesis by increasing pro-lymphangiogenesis-stimulating factors [19, 29]. However, our previous data suggest that excessive accumulation of macrophages in the lymphedematous tissues was accompanied by increased gene expression related to pro-inflammatory macrophages [19]. The excessive infiltration of pro-inflammatory macrophages to the edematous regions of the tail would result in reduced lymphangiogenesis and sustained inflammation and lymphedema [30]. The present study also demonstrated increased gene expressions related to pro-inflammatory macrophages and decreased gene expressions related to reparative macrophages in the macrophages of TP signaling-deficient mice. These results suggest that macrophages lacking TP signaling appeared to exacerbate lymphedema by the accumulation of pro-inflammatory macrophages in edematous tail tissues. Pro-inflammatory macrophages are recruited to the edematous tail tissues after surgery and aggravate lymphedema by inducing IL-6 [31]. In addition, pro-inflammatory macrophages expressing iNOS exacerbated lymphedema and inactivated endothelial NOS, resulting in impaired lymphatic vessel pumping [32]. Furthermore, the present study showed that the CCL2/CCR2 pathway mediated the accumulation of pro-inflammatory macrophages. On the contrary, reparative (anti-inflammatory) macrophages play a role in the promotion of lymphangiogenesis in mouse tail lymphedema models [33]. In addition, reparative macrophages produce VEGF-C to induce secondary lymphedema-induced lymphangiogenesis [33]. Consistent with this, our data suggest that pro-lymphangiogenesis-stimulating cytokines, including VEGF-C and VEGF-D, were produced by TP signaling-induced reparative macrophages. However, the mechanisms by which TP signaling in macrophages modulate macrophage differentiation for the regulation of lymphangiogenesis and lymphedema remain unclear. Taken together, these findings suggest that macrophages contribute to lymphedema development and lymphangiogenesis by producing various pro-/anti-lymphangiogenic growth factors in a context-dependent manner [34].

Our in vivo microscopic studies revealed that exacerbated lymphedema in TP^Δmac^ mice was associated with leakage and stasis of lymphatic fluids in interstitial edematous tissues, suggesting that TP signaling in macrophages affected lymphatic vascular permeability. The lymphatic vascular permeability influences lymphatic transport and drainage of the excessive fluids around the lymphatic vessels. In addition, pro-inflammatory mediators including TNF-α, IL-1β, and IL-6 enhanced the permeability of lymphatic endothelial cell monolayers isolated from rat mesentery [35]. Recently, we reported that lymphatic vessel permeability was enhanced in lymphedematous tissues in this surgically induced tail lymphedema model [19]. The molecular mechanisms responsible for the induction of lymphatic vascular permeability through TP signaling remain unknown and potentially warrant further investigation.

## Conclusions

This study demonstrated that TP signaling attenuates secondary lymphedema and enhances lymphangiogenesis by acting on macrophages. Lymphedema is a significant health problem and affects the quality of life of patients; thus, effective pharmacological treatments are needed. This study suggests that TP signaling may have therapeutic potential for improving lymphedema symptoms by enhancing lymphangiogenesis and lymphatic transport. A better understanding of the interplay between immune cells and lymphatic vessels would offer novel therapeutic opportunities for the prevention of secondary lymphedema.

## Data Availability

The datasets generated during and/or analysed during the current study are available from the corresponding author on reasonable request.

## Abbreviations

AA: Arachidonic acid
BM: Bone marrow
CCL2: C-C motif chemokine 2
CCR2: C-C motif chemokine receptor 2
GAPDH: Glyceraldehyde-3-phosphate dehydrogenase
IL: Interleukin
i.p.: Intraperitoneally
IFN-γ: Interferon-gamma
LYVE-1: Lymphatic vessel endothelial hyaluronan receptor 1
MR: Mannose receptor
PG: Prostaglandin
Prox1: Prospero-related homeobox 1
RT-qPCR: Reverse transcription-quantitative polymerase chain reaction
SD: Standard deviation
TP: Thromboxane prostanoid
TNF-α: Tumor necrosis factor alpha
TXA_2_: Thromboxane A_2_
TXS: Thromboxane synthase
WT: Wild-type

## Declarations

**Ethical approva**l.

The experimental protocols were reviewed by the Institutional Animal Care and Use Committee of the Kitasato University School of Medicine and approved by the Dean of the Kitasato University School of Medicine (Approval no. 2022-060). All experimental studies were performed in accordance with the guidelines of the Science Council of Japan for animal experiment.
